# Complete endocardial cushion defects in pregnancy: a case report

**DOI:** 10.1186/1752-1947-8-91

**Published:** 2014-03-10

**Authors:** Xiangjuan Chen, Biru Xiao, Weiyu Yang, Yunqin Chen, Wenmiao Zhang, Haiyan Zhu

**Affiliations:** 1 Department of Obstetrics and Gynecology, The First Affiliated Hospital of Wenzhou Medical University, 2# Fuxue Road, Wenzhou 325000, People’s Republic of China; 2 Department of Medical Imaging Services, The First Affiliated Hospital of Wenzhou Medical University, 2# Fuxue Road, Wenzhou 325000, People’s Republic of China

**Keywords:** Complete endocardial cushion defect, Eisenmenger’s syndrome, Pregnancy

## Abstract

**Introduction:**

Complete endocardial cushion defect is a congenital heart disease characterized by a variable deficiency of the atrioventricular area in the developing heart. The mortality rate for an unrepaired endocardial cushion defect in pregnancy and the postpartum period is high.

**Case presentation:**

We present a rare case of a pregnant woman with complete endocardial cushion defect. A 20-year-old Chinese woman with unrepaired complete endocardial cushion defect delivered a premature male baby at 33 weeks and six days of pregnancy in our hospital. The baby had a normal human karyotype and a birth defect of hypospadias deformity. Our patient died from heart failure 10 minutes after delivery. She had severe pulmonary hypertension and suspected trisomy 21.

**Conclusion:**

Our experience further emphasizes the necessity of prenatal screening for congenital heart defects and of prompt surgical correction for endocardial cushion defects during infancy. Mortality for endocardial cushion defect during pregnancy and the postpartum period is high and women with complete endocardial cushion defect should avoid pregnancy, especially those women who cannot intellectually judge their risks.

## Introduction

A complete endocardial cushion defect (ECD) involves a large primum atrial septal defect and an inlet ventricular septal defect of variable size. Additionally, there is only one large heart valve (common valve) instead of two distinct atrioventricular valves (mitral and tricuspid). An ECD occurs early in fetal development, presumably due to a failure of the endocardial cushions to facilitate normal development of each septum and the atrioventricular valves. Surgery is usually performed between three and six months of age depending upon severity. A complete ECD represents approximately 3% of congenital heart defects [1]. It is thought that more than 50% of complete ECDs occur in conjunction with trisomy 21 [1]. However, Ashok *et al*. reported that an ECD often occurs with other cardiac or extracardiac abnormalities but seems to be less frequently associated with chromosomal abnormality trisomy 21 in the population of South India [2]. The cause of an ECD remains unclear. Agopian *et al*. reported that maternal pregestational diabetes and obesity are significantly associated with a non-symptomatic complete ECD in the fetus [3].

Intracardiac shunting often occurs in patients with ECDs. Initially, blood usually flows from left to right, because the pressure on the left side of the heart is higher than that on the right side. As the left-to-right shunting is prolonged, continuous exposure of the pulmonary vasculature to systemic arterial pressure leads to progressive changes in the pulmonary microvasculature, causing increased pulmonary vascular resistance and pulmonary hypertension. When pressure in the pulmonary circulation exceeds systemic pressure, blood flows from right to left as well, resulting in bidirectional intracardiac shunting. This condition is also called Eisenmenger’s syndrome (ES). The right-to-left shunting can significantly dilute well-oxygenated blood, resulting in cyanosis. Patients with ES usually present with symptoms such as breathlessness, fatigue, chest pain and syncope [4]. The pulmonary hypertension occurring in ES imposes serious risks during pregnancy and the postpartum period. The decreased systemic vascular resistance associated with pregnancy worsens the right-to-left shunting, leading to a deterioration of cyanosis. Gleicher *et al*. reported that the mortality rate for pregnancy in ES is 52% [5]. The majority of maternal deaths occur during the first week after delivery. Cesarean sections and other operations are associated with extremely high maternal mortality during pregnancy. The maternal mortality rate of pregnancy with ES is reported to be as high as 50% to 65% with Cesarean section [6]. At least 54.9% of deliveries occur prematurely [5]. Low birth weight can be as high as 26% [5].

Echocardiography is an effective diagnostic approach for an ECD and the diagnosis of a complete ECD at prenatal stage is usually very accurate. Smrcek *et al*. demonstrated that the success rate for a diagnosis of fetal congenital heart abnormalities using echocardiography is 45% at 11 weeks, 90% at 12 to 14 weeks, and 100% at 15 weeks [7]. In this report, we present a rare case of pregnancy in a patient with a complete ECD.

## Case presentation

A 20-year-old Chinese woman first visited our hospital when she was 28 weeks pregnant. She was mentally retarded with typical features of trisomy 21. She had regular menstrual cycles before pregnancy. She had dyspnea, chest pain and fatigue with mild physical exertion. She also had trouble lying down in a supine position.

During the examination, her electrocardiogram showed sinus tachycardia and right atrial and right ventricular hypertrophy. Her heart rate was 115 beats per minute. Her symptoms were suggestive of an unrepaired ECD. She had severe left and right common valve insufficiency. Color flow imaging and pulsed wave Doppler demonstrated bidirectional cardiac shunting with dominant left-to-right shunting. Her left and right heart pressures were nearly equal. Based upon the natural history of an unrepaired ECD, we suspected that our patient had severe pulmonary hypertension.

Because of the extreme high risk of mortality for our patient, the obstetrician, cardiologist and anesthesiologist of our hospital offered to terminate the pregnancy. Our patient’s family refused the advice and insisted on continuing the pregnancy. Our patient was also advised to stay in our hospital for close monitoring and proper treatment, and again her family refused our advice.

Our patient returned at 33 weeks and six days of pregnancy because of lower abdominal pain accompanied with vagina fluid outflow for two hours. Her physical examination on admission showed a temperature of 35°C, pulse of 130 beats per minute, blood pressure at 140/80mmHg, respiration 33 breaths per minute (provided with 5L oxygen per minute), severe dyspnea, cyanosis, lower extremity edema, grade three to six heart murmur at her left intercostal space, and grade two to three systolic murmur at the apex of her heart. Her oxygen saturation was 19.4%.

Her baby was in breech presentation. The fetal heart rate was 120 beats per minute. Our patient had regular uterine contractions with an interval of three to four minutes. Her cervical dilation was 10cm with rupture of the fetal membranes. An ultrasound showed that her fetus was in the left sacral anterior position with a biparietal diameter of 72mm and femur length of 56mm. The amniotic fluid index was 20mm. An analysis of the fetal blood gas showed 48.8mmHg carbon dioxide and 22.1mmHg oxygen, with pH 7.15. A male baby was delivered by gentle traction. The weight of the baby was 1110g. His one-minute Apgar score was 5. His five-minute Apgar score was 10. Our patient died 10 minutes after delivery. When her heart stopped beating, standard cardiopulmonary resuscitation was performed. Our patient was not on a ventilator. The premature infant had a normal human karyotype and had intrapartum asphyxia and hypospadias deformity. The baby was treated at the Neonatology department of our hospital and discharged two months later with a body weight of 2055g. The baby does not have congenital heart disease.

## Discussions

Progressive pulmonary complications and congestive heart failure are commonly seen in infancy. Fifty percent of infants with an untreated complete ECD, whether symptomatic or non-symptomatic, will die during the first year of life [8]. Surgical treatment is preferably scheduled before six to twelve months of life [8]. Without early surgical intervention, only approximately 4% of patients with a complete ECD survive to five years of age [8]. Surgical repair in early infancy is usually effective. Most children can lead happy and productive lives after surgical repair. Our patient’s mother did not have fetal echocardiography when she was pregnant. Thus, our patient was born with a complete ECD, and her condition was left untreated due to poverty. Our patient was in severe chronic heart failure through a combination of her heart disease and severe pulmonary hypertension.

A woman’s cardiac output during labor increases 45% compared to that prior to pregnancy [9]. Approximately 300mL of blood enters the systemic circulation during each episode of uterine contraction. The pain caused by uterine contraction substantially escalates the blood pressure and heart rate. After the placenta is delivered, the uteroplacental vascular bed contracts, resulting in 500mL of blood entering the mother’s systemic circulation. All these factors increase the burden of the heart. The risk of mortality for patients with cyanotic heart disease reaches its peak during labor and puerperium because of the following: blood loss during labor, reduced venous return caused by vena cava syndrome, reduced resistance of the systemic circulation, and increased resistance in pulmonary vessels caused by micro-thrombosis or hypoxemia. All those conditions can increase right-to-left cardiac shunting. For our patient, we suspect the cause of death was heart failure. Our patient’s heart, which was already malfunctioning due to the complete ECD, could not tolerate a sudden increase in the volume of blood returning to her heart after delivery. Her condition escalated very quickly after delivery, and she died 10 minutes after childbirth. Cardiopulmonary resuscitation failed. Yentis *et al*. compared the maternal mortality of patients with ES from 1990 to 1995 with the initial reports from the 1950s, and found that maternal mortality associated with ES has remained unchanged over the past 50 years [10]. Thus, women with ES should avoid pregnancy, and early termination of the pregnancy should be strongly recommended if a patient with ES does become pregnant. When a patient chooses to continue the pregnancy, a multidisciplinary team including obstetrician, cardiologist, anesthesiologist, neonatologist and intensive care physician should coordinate the management of the patient.

## Conclusion

We emphasize the importance of prenatal screening for congenital heart defects and prompt surgical correction for infants with an ECD. The mortality rate for an ECD during pregnancy and the postpartum period is high. Thus, women with a complete ECD should avoid pregnancy, especially those women who cannot intellectually judge their risks.

## Consent

Written informed consent was obtained from the patient and patient’s family for publication of this case report and any accompanying images. A copy of the written consent is available for review by the Editor-in-Chief of this journal.

## Abbreviations

ECD: complete endocardial cushion defect; ES: Eisenmenger’s syndrome.

## Competing interests

The authors declare that they have no competing interests.

## Authors’ contributions

XC drafted the manuscript. BX, WY, YC and WZ collected the clinical data. HZ proposed the study and helped to draft the manuscript. All authors read and approved the final manuscript.

## References

[B1] Calabrò R Limongelli G Complete atrioventricular canal Orphanet J Rare Dis 2006 1 8 12 10.1186/1750-1172-1-8 16722604 PMC1459121

[B2] Ashok M Thangavel G Indrani S Suresh S Atrioventricular septal defect–associated anomalies and aneuploidy in prenatal life Indian Pediatr 2003 40 659 664 12881623

[B3] Agopian AJ Moulik M Gupta-Malhotra M Marengo LK Mitchell LE Descriptive epidemiology of non-syndromic complete atrioventricular canal defects Paediatr Perinat Epidemiol 2012 26 515 524 10.1111/ppe.12006 23061687 PMC3482101

[B4] Naguib MA Dob DP Gatzoulis MA A functional understanding of moderate to complex congenital heart disease and the impact of pregnancy. Part II: tetralogy of Fallot, Eisenmenger’s syndrome and the Fontan operation Int J Obstet Anesth 2010 19 306 312 10.1016/j.ijoa.2009.10.009 20627686

[B5] Gleicher N Midwall J Hochberger D Jaffin H Eisenmenger’s syndrome and pregnancy Obstet Gynecol Surv 1979 34 721 741 10.1097/00006254-197910000-00001 503376

[B6] Makaryus AN Forouzesh A Johnson M Pregnancy in the patient with Eisenmenger’s syndrome Mt Sinai J Med 2006 73 1033 1036 17195894

[B7] Smrcek JM Berg C Geipel A Fimmers R Diedrich K Gembruch U Early fetal echocardiography: heart biometry and visualization of cardiac structures between 10 and 15 weeks’ gestation J Ultrasound Med 2006 25 173 182 16439780 10.7863/jum.2006.25.2.173

[B8] Berger TJ Blackstone EH Kirklin JW Survival and probability of cure without and with surgery in complete atrioventricular canal Ann Thorac Surg 1979 27 104 111 10.1016/S0003-4975(10)63249-3 453968

[B9] Hunter S Robson SC Adaptation of the maternal heart in pregnancy Br Heart J 1992 68 540 543 10.1136/hrt.68.12.540 1467047 PMC1025680

[B10] Yentis SM Steer PJ Plaat F Eisenmenger’s syndrome in pregnancy: maternal and fetal mortality in the 1990s Br J Obstet Gynaecol 1998 105 921 922 10.1111/j.1471-0528.1998.tb10240.x 9746388

